# Methodological challenges surrounding QALY estimation for paediatric economic evaluation

**DOI:** 10.1186/s12962-022-00345-4

**Published:** 2022-03-03

**Authors:** Stavros Petrou

**Affiliations:** Nuffield Department of Primary Care Health Sciences, University of Oxford, Radcliffe Observatory Quarter, Woodstock Road, Oxford, OX2 6GG UK

**Keywords:** QALYs, Quality-adjusted life years, Paediatrics, Childhood, Methods, Valuation

## Abstract

Cost-utility analysis remains the preferred form of economic evaluation for health technology assessment, pricing and reimbursement authorities in several countries. The results of cost-utility analyses are commonly expressed in terms of incremental cost per quality-adjusted life year (QALY) gained where the QALY combines length of life and health-related quality of life in a single metric. This commentary provides an overview of key methodological challenges surrounding QALY estimation for paediatric economic evaluation. These challenges include issues surrounding the relevant attributes to incorporate into measurement instruments, appropriate respondents for the measurement and valuation tasks, perspectives adopted when completing valuation tasks, potential sources of bias in the description and valuation processes, and the paucity of psychometric evidence for existing measures. In addition, the commentary considers methodological challenges raised by research aimed at assessing whether a QALY gain by a child should be valued equally to a QALY gain by an adult.

## Introduction

Economic evaluation involves the comparative analysis of alternative programmes or interventions in terms of their costs and consequences [1]. Cost-utility analysis remains the preferred form of economic evaluation for health technology assessment (HTA), pricing and reimbursement authorities in several countries, including the Pharmaceutical Benefits Advisory Committee (PBAC) in Australia [2], the Canadian Agency for Drugs and Technologies in Health (CADTH) in Canada [3], the National Institute of Health and Care Excellence (NICE) in England and Wales [4], and the Scottish Medicines Consortium (SMC) in Scotland [5]. The results of cost-utility analyses are commonly expressed in terms of incremental cost per quality-adjusted life year (QALY) gained where the QALY combines length of life and health-related quality of life in a single metric [6]. For government agencies, the QALY has the advantage of facilitating comparisons of health outcomes across different health care interventions for disparate health conditions. It offers an additional advantage in that the approaches to valuing health-related quality of life outcomes, typically on a cardinal scale anchored at zero (representing death) and one (representing full health), capture people’s preferences for outcomes beyond those framed by a narrow biomedical perspective.

Health economists have developed a number of approaches for the measurement and valuation of preference-based health-related quality of life outcomes (or health utilities) for inclusion within the QALY metric. These include direct valuation methods using scaling techniques, such as the standard gamble (SG) and time trade-off (TTO) approaches, where the measurement and valuation of preferences occur in a single step [6]; health rating scales, such as the Visual Analogue Scale [1]; multi-attribute health status classification systems with preference scores, such as the EQ-5D [7], Health Utilities Index (HUI) [8], SF-6D [9], Quality of Well-Being Scale [10] and Assessment of Quality of Life (AQoL or AQoL-5D) [11]; mapping from non-preference-based measures onto generic preference-based measures of health [12]; and the development of *de novo* measures [13]. HTA agencies in several jurisdictions provide guidance on their preferred approach for the measurement and valuation of preference-based health-related quality of life outcomes for QALY estimation. In Canada, for example, CADHT accepts both the HUI Mark 2 and HUI Mark 3 as multi-attribute health status classification systems with preference scores for their reference case despite their differing attributes [3], whilst the EQ-5D is the preferred measure of the preference-based health-related quality of life outcomes of adults in England and Wales [4] and in Scotland [5]. In Australia, PBAC remains broadly agnostic regarding a preferred approach for the measurement and valuation of preference-based health-related quality of life outcomes, although a steer is provided to ensure that utility weights are applicable to the general Australian population [2]. Even in jurisdictions with a clearly preferred approach, there is recognition that a ubiquitous approach to the measurement and valuation of preference-based health-related quality of life outcomes may be inappropriate; for example, if there is qualitative empirical evidence on the lack of content validity for a preferred measure or if the preferred measure performs poorly in tests of construct validity and responsiveness in a particular population [4].

## Methodological challenges surrounding measurement and valuation of health utilities in the paediatric context

The measurement and valuation of preference-based health-related quality of life outcomes for QALY estimation raises particularly acute methodological challenges in paediatric populations. Methodological concerns that are specific to childhood and adolescent populations include issues surrounding the relevant attributes to incorporate into measurement instruments, appropriate respondents for the measurement and valuation tasks, potential sources of bias in the description and valuation processes, and the paucity of psychometric evidence for existing measures [14]. These limitations have been mitigated to a degree by the development of generic childhood and adolescent-specific multi-attribute health status classification systems that generate preference-based scores. A previous review article [15] identified nine such measures validated for use across health conditions in mid and/or late childhood or in adolescence, namely the HUI Mark 2 [8], HUI3 Mark 3 [8], Child Health Utility 9D (CHU9D) [16], Assessment of Quality of Life 6 Dimension (AQoL-6D) [17], 16D [18], 17D [19], EQ-5D-Y [20], Quality of Well-Being scale (QWB) [21] and Assessment of Health Utility Measurement (AHUM) [22]. Furthermore, research is ongoing to develop multi-attribute health status classification systems that generate preference-based scores for infancy [23] or targeted at specific childhood conditions, such as excess weight [24] or oral health [25]. A recent review of PBAC public summary documents in Australia that considered funding decisions around medicines used by children found that decision-making uncertainty would have been reduced or potentially reduced for approximately 85% of the medicines considered if generic childhood and adolescent-specific multi-attribute health status classification systems that generate preference-based scores had been available and/or used [26].

Each of the generic childhood and adolescent-specific multi-attribute health status classification systems that generate preference-based scores differ in choice of attributes or domains and their conceptual underpinnings, valuation protocol, choice of informant, perspective adopted by the respondent, appropriateness for each developmental stage of childhood and adolescence, and formatting and mode of administration, which is likely to independently impact on the health utility values that are generated. The domain coverage of the Infant health-related Quality of Life Instrument (IQI) includes sleeping, feeding and breathing [23], whilst that for the AHUM includes concerns facing adolescents such as self-image and health perceptions [22]. The choice of informant for the task of describing the child’s health status should take account of poor inter-rater agreement between children’s self-reports and parent-proxy reports for subjective attributes such as cognition, emotion and pain that have been identified by many studies [27]. Furthermore, the development of generic childhood and adolescent-specific multi-attribute health status classification systems that generate preference-based scores raises the normative question of whose values should underpin the preference weights for paediatric health states. Many health economists argue that representative samples of the general population should be asked to act as the social decision-maker when eliciting preferences for health states that can inform cost-effectiveness based decision-making [28]. Recent research has explored whether adult and adolescent preferences differ when asked to value EQ-5D-Y-3 L health states [29, 30]. In practice, however, samples of adults have largely been used to derive value sets for generic childhood and adolescent-specific multi-attribute health status classification systems that generate preference-based scores [15, 31]. This raises the question of which perspective they should adopt when asked to value a childhood or adolescent health state. Adopting different perspectives, for example that of a hypothetical child, themselves as a child, themselves at the current age but experiencing that state, or that of another adult experiencing that state, can affect their preference structures [30, 32]. Arguably, there is scope for incorporating the preferences of individuals generally excluded from social decision-making deliberations, such as of children, into the processes of generating the value sets for generic childhood and adolescent-specific multi-attribute health status classification systems that generate preference-based scores. However, even if it’s accepted that children’s values are valid, approaches for capturing those values have been stymied by tools that require developed cognitive abilities and linguistic skills and articulation of preferences for health states using complex concepts of gain and loss in health economic terms [33]. Their values might also be influenced by specific design features of the preference elicitation task, for example, the valuation protocol, mode of administration or method of anchoring on the 0–1 (death to full health) utility scale [14]. Recent interest has considered the use of mixed samples of adolescents and adults to value child and adolescent health states [34].

None of the generic childhood and adolescent-specific multi-attribute health status classification systems that generate preference-based scores is validated for use across all stages of childhood and adolescence, which raises concerns over comparability of their value sets. Furthermore, attempts at mapping between these value sets have been limited to a small number of measures applied in narrow age banded groups [35]. Particular concerns arise when utility values generated by these measures act as inputs into economic evaluations with time horizons extending across the life course. Paediatric evaluations that adopt a life-time horizon typically value health states using a common measure or valuation protocol [36], overlooking the inherent methodological limitations of this approach. Ultimately, methodological guidance from health technology assessment agencies may be required to inform preferred measures that should be applied at different stages of life, supplemented by evidence from mapping studies that accurately predict the relationships between those measures.

## Should a QALY gain by a child be valued equally to a QALY gain by an adult?

Beyond challenges surrounding methods for measuring and valuing health utilities in or on behalf of children and adolescents, a separate set of challenges surround whether overall measures of health consequence, such as an additional QALY, should be valued equally across childhood, adolescent and adult populations. The revealed preference literature has generated evidence that indicates that the value individuals place on reducing health risks or achieving health gains, usually expressed in terms of a monetary value of statistical life, is higher for children than for adults [37]. However, the values estimated in the revealed preference literature are largely based on choices made by parents on the part of family members and, consequently reflect, at least in part, altruistic concerns. Furthermore, revealed preference studies tend to be based on evidence from individuals who are poorly informed about the differential health risks associated with the choices they face. They also provide limited evidence for decisions made in health care systems where care is provided at zero or subsidised prices at the point of use. Perhaps more pertinent evidence for those concerned with deriving social, as opposed to individual, values for age weights for QALYs is provided by stated preference studies. Notably, stated preference studies in the health context provide some evidence of an inverted-U shaped relation between age and the value placed on health gain [37, 38], consistent with the relationship between age and the value of statistical life identified in labour market studies [39]. A large stated preference study in this area surveyed a nationally representative sample (n = 587) of the population in England using two preference elicitation techniques, a discrete choice experiment and a ‘matching’ (or person trade-off) approach. The former revealed that age did not have a strong impact on respondents’ choices over and above the health (QALY) gains presented, while the latter revealed a general tendency to give greater weight to 20- to 40-year olds over other age groups (0- to 20-, 40- to 60- and 60- to 80-year olds) [40, 41]. More recent research in this area has considered age together with broader distributional concerns, for example socioeconomic status or baseline severity of illness, for priority setting [42]. Notably, however, there is no consensus around a number of methodological features of stated preference studies that aim to generate age weights for QALYs, including source(s) for preferences, appropriate dimensions for the health-related quality of life component of the QALY measure, preferred valuation protocol, and methods for controlling for context and design effects on derived values. Moreover, there are operational concerns surrounding the application of age weights for QALYs within economic evaluations that extend in time horizon across the life course. In particular, systems that rely on a cost-effectiveness threshold to inform decision-making will ultimately need to consider how they apply age-related weights to the health benefits foregone as a result of displaced activities [37].

## Conclusions

In conclusion, this paper has provided an overview of key methodological challenges surrounding QALY estimation for paediatric economic evaluation. This is in area of active research enquiry, for example, by the TORCH (https://torch.hykecreative.com.au) and QUOKKA (https://quokkaresearchprogram.org) research programmes in Australia. These research programmes are assessing the relative merits of alternative measurement and valuation approaches, developing new preference-based value sets for existing generic childhood and adolescent-specific measures, and assessing the comparability of QALYs across childhood and adolescent populations. Ultimately, this evidence should generate methodological advancements that inform cost-effectiveness based decision making by health technology assessment, pricing and reimbursement authorities.

## Data Availability

Not applicable.
